# Lower tidal volume at initiation of mechanical ventilation may reduce progression to acute respiratory distress syndrome: a systematic review

**DOI:** 10.1186/cc11936

**Published:** 2013-01-18

**Authors:** Brian M Fuller, Nicholas M Mohr, Anne M Drewry, Christopher R Carpenter

**Affiliations:** 1 Department of Anesthesiology, Division of Critical Care, Division of Emergency Medicine, Washington University in St. Louis School of Medicine, 660 South Euclid Avenue, St. Louis, MO, USA; 2 Department of Emergency Medicine, Department of Anesthesia, Division of Critical Care, Roy J. and Lucille A. Carver College of Medicine, University of Iowa, 375 Newton Road, Iowa City, IA, USA; 3 Department of Anesthesiology, Division of Critical Care, Washington University in St. Louis School of Medicine, 660 South Euclid Avenue, St. Louis, MO, USA; 4 Division of Emergency Medicine, Washington University in St. Louis School of Medicine, 660 South Euclid Avenue, St. Louis, MO, USA

## Abstract

**Introduction:**

The most appropriate tidal volume in patients without acute respiratory distress syndrome (ARDS) is controversial and has not been rigorously examined. Our objective was to determine whether a mechanical ventilation strategy using lower tidal volume is associated with a decreased incidence of progression to ARDS when compared with a higher tidal volume strategy.

**Methods:**

A systematic search of MEDLINE, EMBASE, CINAHL, the Cochrane Library, conference proceedings, and clinical trial registration was performed with a comprehensive strategy. Studies providing information on mechanically ventilated patients without ARDS at the time of initiation of mechanical ventilation, and in which tidal volume was independently studied as a predictor variable for outcome, were included. The primary outcome was progression to ARDS.

**Results:**

The search yielded 1,704 studies, of which 13 were included in the final analysis. One randomized controlled trial was found; the remaining 12 studies were observational. The patient cohorts were significantly heterogeneous in composition and baseline risk for developing ARDS; therefore, a meta-analysis of the data was not performed. The majority of the studies (*n *= 8) showed a decrease in progression to ARDS with a lower tidal volume strategy. ARDS developed early in the course of illness (5 hours to 3.7 days). The development of ARDS was associated with increased mortality, lengths of stay, mechanical ventilation duration, and nonpulmonary organ failure.

**Conclusions:**

In mechanically ventilated patients without ARDS at the time of endotracheal intubation, the majority of data favors lower tidal volume to reduce progression to ARDS. However, due to significant heterogeneity in the data, no definitive recommendations can be made. Further randomized controlled trials examining the role of lower tidal volumes in patients without ARDS, controlling for ARDS risk, are needed.

2013 Fuller *et al*.; licensee BioMed Central Ltd. This is an open access article distributed under the terms of the Creative Commons Attribution License (http://creativecommons.org/licenses/by/2.0), which permits unrestricted use, distribution, and reproduction in any medium, provided the original work is properly cited.

## Introduction

Close to 200,000 cases of acute lung injury (ALI) or acute respiratory distress syndrome (ARDS) occur annually in the United States [1,2]. It remains a leading cause of death in critically ill patients, with a mortality rate of approximately 30%, but as high as 60% in the elderly [1,3,4]. ARDS also represents a large societal burden, accounting for 3.6 million hospital days annually and significant long-term sequelae in survivors [1,3]. These include various neuropsychological impairments (e.g. depression, cognitive decline), persistent weakness and pulmonary dysfunction, and decreased quality of life [3,5].Unfortunately, the number of ARDS trials demonstrating improved clinical outcomes are far outnumbered by those that have not [6-21]. In patients with ARDS, the current body of evidence supports a lower tidal volume strategy as best practice [6].

In contrast, the optimal tidal volume in patients without ARDS is uncertain [22,23]. Historically, it was thought that high tidal volume not only was safe, but also was preferred to avoid the hypoxia, atelectasis, and acidosis associated with mechanical ventilation with lower tidal volumes [24-26]. This led to the common use of tidal volumes in the 12- to 15-ml/kg range [27]. However, experimental data suggest that tidal volume is a major contributor to the development of lung injury [28]. Clinical data in intensive care unit (ICU) patients also suggests that larger tidal volume may contribute to the development of ARDS [29-31], suggesting that this syndrome may in part be preventable. However, this evidence has been inconclusive, because of observational trial design, small sample sizes, and lack of reproducibility in different patient populations [32,33]. In patients without lung injury at the time of endotracheal intubation, progression to ARDS can occur over the course of hours and may increase mortality by >30% [30,34,35]. This has led to increased interest in ARDS prevention and the examination of a lower tidal volume strategy to prevent its development in patients without lung injury.

We therefore aimed to systematically review the evidence regarding the role of tidal volume as a contributor to ARDS progression in patients without ARDS. The objectives of this study were to perform a systematic review of the literature comprising a comprehensive search strategy and standardized analysis techniques to determine whether lower tidal volumes, compared with higher tidal volumes, are associated with a decreased incidence of ARDS in mechanically ventilated patients. We hypothesized that lower tidal volume ventilation is associated with a decrease in progression to ARDS in mechanically ventilated patients, but the existing literature lacks the methodologic quality to provide definitive recommendations for the best tidal volume in patients without ARDS.

## Materials and methods

This systematic review was designed, conducted, and reported in accordance with the Preferred Reporting Items for Systematic Reviews and Meta-Analysis (PRISMA) and Meta-analysis of Observational Studies in Epidemiology (MOOSE) guidelines [36,37]. It did not require ethical approval from Washington University Human Research Protection Office. It is recognized that ALI and ARDS have undergone recent definitional change [38]. Although the definitional criteria in place during the systematic search for clinical trials, as well as the conduct of each study, was the American-European Consensus Conference (AECC) criteria, for consistency in reporting according to the new Berlin definition, we use the term ARDS to refer to both ARDS and ALI (as reported in previous literature) [2,38].

### Search for and identification of studies

We followed a written protocol (see Additional file 1) that was finalized before beginning the search. We initiated our timeline from 1967 (first description of ARDS) through 2011 and searched MEDLINE, EMBASE, CINAHL, and the Cochrane Library, by using a combination of standardized search terms and keywords covering the basic concepts of *acute lung injury, acute respiratory distress syndrome, mechanical ventilation, ventilator-induced lung injury, prevention, outcomes, and clinical trial*. A trained information professional experienced in systematic reviews assisted in designing the search strategy and in conducting the search. Two authors (BMF, NMM) also manually screened the reference lists of the articles selected for inclusion to identify additional studies. To identify potential unpublished data from clinical trials that have completed enrollment, BMF and NMM also (a) manually searched abstracts from the Society of Critical Care Medicine, European Society of Intensive Care Medicine, American Society of Anesthesiology, American Thoracic Society, CHEST, and the Society of Academic Emergency Medicine from 2008 to 2011; and (b) searched online for details of clinical trials registration (http://ClinicalTrials.gov). BMF also contacted principal investigators of published and unpublished studies for clarification of potential data for inclusion, as needed.

### Inclusion criteria

Studies were eligible for review regardless of language or publication type. We included randomized controlled trials, prospective and retrospective cohort analyses, cross-sectional studies, and before-and-after trials of adults (older than 17 years) undergoing invasive positive-pressure ventilation without ARDS at the time of initiation of mechanical ventilation, and in which tidal volume was independently studied (either retrospectively or prospectively) as a predictor variable for outcome. We excluded studies involving patients with established ARDS by consensus criteria [2], studies of nonintubated patients (e.g. noninvasive positive-pressure ventilation), studies of one-lung ventilation, studies in which tidal volume was not the only ventilator variable studied or manipulated (e.g. tidal volume and positive end-expiratory pressure), and studies in which the development of ARDS was not a primary or secondary outcome measure. We also excluded articles that were reviews, correspondence, editorials, and nonhuman studies. We screened the reference list of all review articles to identify additional studies for inclusion. Corresponding authors were contacted, where appropriate, for clarification of data or study type.

### Study selection and data abstraction

Two reviewers (BMF and NMM) independently screened the titles and abstracts of identified studies for potential eligibility. After this relevance screen, full text articles were assessed for eligibility, and the two reviewers compared their exclusion logs to determine whether there was disagreement. All studies deemed potentially relevant after the screen were obtained, and the full manuscripts were reviewed for further exclusion. In cases of disagreement, a third reviewer (AD) assessed the study, and a consensus was reached between the three reviewers. Any disagreements in this collection were resolved with a consensus among the reviewers.

### Assessment of study quality

We assessed the quality of clinical trials selected for inclusion by using the Cochrane Collaboration's tool for assessing the risk of bias in clinical trials, evaluating four domains: random sequence generation, concealment of allocation, blinding, and selective outcome reporting [39]. High quality was defined as a grade of A in at least three of the four methodology domains. For studies of observational design, each was assessed for its reporting of adherence to the Strengthening the Reporting of Observational Studies in Epidemiology (STROBE) Statement [40].

### Assessment of publication bias

Because detecting publication bias is difficult, we sought to minimize bias through a medical librarian assisted comprehensive search strategy and the use of study registries. We planned to use a graphic display (funnel plot) of the size of the treatment effect against the precision of the trial to evaluate for potential publication bias [41].

### Data analysis

Our original intent, after conducting the systematic review, was to conduct a meta-analysis of the data by using Review Manager (RevMan, Version 5.1, Copenhagen: The Nordic Cochrane Centre, The Cochrane Collaboration, 2011). However, because of qualitative heterogeneity between the studies with respect to the patient populations studied and the baseline risk for ARDS development, this was not possible. RevMan was used to generate the funnel plot.

## Results

### Search and selection

**Figure 1:**
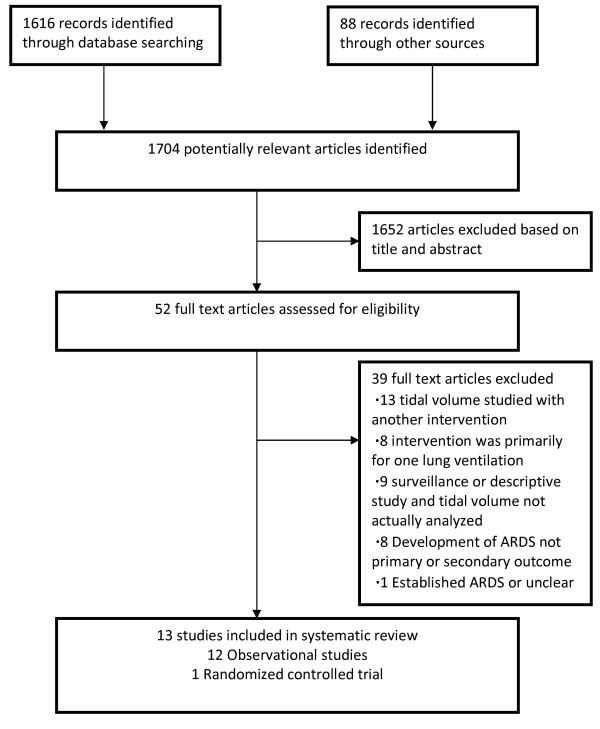
**Search, inclusion, and exclusion flow diagram**.

The comprehensive search yielded a total of 1,704 potentially relevant publications. Details regarding the search, study selection, and reason for exclusion are shown in Figure 1.

### Inclusion

After the relevance search, a complete manuscript review was performed on the remaining 52 articles. Thirteen studies were included in the final analysis [29-31,34,35,42-49]. The characteristics of the included studies are shown in Table 1.

**Table 1:** Study characteristics

Randomized controlled trial								
**Author, year**	***n***	**Intervention**	**Location**	**Primary patient population**	**High-quality RCT?^a^**	**Primary outcome**	**Secondary outcome**	**Comments^b^**

Determann, 2010	150	VT 6 ml/kg PBW vs. 10 ml/kg PBW	Medical/surgical ICU	Cardiac arrest, neurologic disease	YES	Cytokine levels in BAL and plasma	Development of ARDS	Could be randomized up to 36 hours after mechanical ventilation

**Observational studies**								

**Cohort studies **								

**Author, year**	***n***	**Study design**	**Location **	**Primary patient diagnoses**	**High quality^c^**	**Primary outcome**	**Secondary outcomes**	**Comments**

Gajic, 2004	332	Retrospective	Medical/surgical ICUs	Shock, sepsis	NO	Development of ARDS	Hospital mortality, ventilator-free days	Excluded patients ventilated <48 hours

Gajic, 2005	3,261	Retrospective	Multicenter registry of medical/surgical ICUs	Postoperative, coma, pneumonia	NO	Development of ARDS	None mentioned	Excluded patients ventilated <48 hours

Kahn, 2006	620	Retrospective	Neuro ICU	Aneurysmal subarachnoid hemorrhage	NO	Development of ARDS	Incidence of lung-protective ventilation	Tidal volume obtained from author contact.

Mascia, 2007	82	Prospective	Four European ICUs	Severe traumatic brain injury	NO	Development of ARDS	ICU LOS, VF days, ICU mortality	Excluded patients with ARDS <24 hours from admission

Yilmaz, 2007	375	Prospective, before-after	Medical/surgical ICU	Sepsis, pneumonia	NO	Development of ARDS	Hospital mortality, duration of ventilation, ICU LOS, VF days	Excluded patients ventilated <48 hours

Plurad, 2007	2,346	Retrospective	Surgical ICU	Trauma	NO	Development of late ARDS	Not mentioned	Defined late ARDS as >48 hours after admission

Iscimen, 2008	160	Prospective	Medical ICU	Septic shock	NO	Development of ARDS	Hospital mortality, hospital length of stay	

Jia, 2008	789	Retrospective	Medical/surgical/cardiac ICUs	Unclear	NO	Development of ARDS	Not mentioned	Excluded patients ventilated <48 hours. 341 patients had data missing on VT/PBW

Pasero, 2008	200	Prospective	Cardiac ICU	Patient status after cardiopulmonary bypass	NO	Development of ARDS	Not mentioned	Abstract only

Hughes, 2010	89	Retrospective	OR	Abdominal, orthopedic, vascular surgery within 24 hours of ICU admit	NO	Development of ARDS	Not mentioned	Assessed outcomes for 7 days. Excluded many risks for ARDS development

Blum, 2011	53910	Retrospective	OR: all noncardiothoracic and nontransplant procedures	ASA I, II	NO	Development of ARDS	Not mentioned	Abstract only

**Case-control study**								

**Author, year**	***n***	**Study design**	**Location **	**Primary patient diagnoses**	**High quality**	**Primary outcome**	**Secondary outcomes**	**Comments**

Fernandez-Perez, 2009	4,420	Prospective, nested	OR with general anesthesia for ≥3 hours	Elective operations	NO	Postoperative respiratory failure due to ARDS	Length of stay, 60-day survival, 1-year survival	Excluded patients with "prevalent" risk factors for ARDS. 1^st ^hour ventilator variables were primary predictor variable

^a^As assessed by Cochrane Collaboration's tool for assessing risk of bias in clinical trials. Was assigned a grade of "A" (high quality) in the following methodology domains: random-sequence generation, concealment of allocation, and selective outcome reporting; assigned a grade of "C" (unknown) in the following methodology domain: blinding. To explain, for trials in which blinding is not feasible at the point of intervention (such as a tidal volume trial), a grade of "A" would be assigned if the investigator collecting the primary outcome were blinded to the treatment allocation. ^b^For the RCT, there was no reported assessment of adherence to the ventilator/intervention protocol. ^c^As assessed by STROBE guidelines. ARDS, acute respiratory distress syndrome; ASA, American Society of Anesthesiologists; BAL, bronchoalveolar lavage; ICU, intensive care unit; LOS, length of stay; PBW, predicted body weight; VF, ventilator free; VT, tidal volume.

### Study characteristics and outcomes reporting

The 13 studies were published over a 7-year period (2004 through 2011). One study was a randomized controlled trial (RCT), and 12 were of observational trial design. On the methodologic quality assessment, the RCT was rated as high quality by Cochrane Collaboration's tool for assessing the risk of bias in clinical trials. In this trial, Grade A was given for random-sequence generation (sequence adequately generated), concealment of allocation (performed concealment allocation), and selective outcome reporting (free of suggestion of selective outcome reporting). For clinical trials in which blinding is not feasible at the point of intervention (potential methodologic issue for mechanical ventilation trials), high quality can still be assigned if the investigator collecting the primary outcome is blinded to the treatment allocation. This was unclear in the included RCT; therefore, a grade C was given for blinding (unknown whether investigators collecting the primary outcome were blinded to allocation). None of the 12 observational trials reported any adherence to the STROBE guidelines [40]. Two of the trials were multicenter studies (one retrospective registry and one prospective observational trial), and 11 were performed at a single center. Ten trials involved interventions and patients in medical, surgical, neurologic, trauma, or cardiac ICUs. Three trials were from the operating room. No studies examined mechanical ventilation in the Emergency Department (ED). Two of the studies were presented in abstract form only.

**Figure 2:**
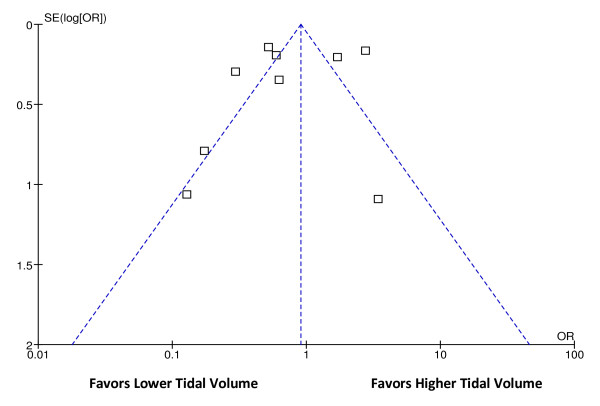
**Funnel plot for assessment of publication bias**.

Significant heterogeneity was found with respect to the patient population studied and the diagnoses leading to respiratory failure (Table 1). Examples include cardiac arrest, neurologic disorders such as subarachnoid hemorrhage and traumatic brain injury, postoperative patients, sepsis, and elective surgical patients. Visual inspection of the funnel plot (Figure 2) indicated potential publication bias, because observed asymmetry suggested unpublished research that favored not using low tidal volumes [41]. Although ARDS was defined according to the AECC criteria in each of the studies, differences existed in given criteria between studies [2]. For example, two studies required the criteria to be fulfilled for 2 consecutive days [29,45]; one study required criteria for 24 hours [47]; one study required criteria for three consecutive arterial blood gases [31]; and one study allowed the criterion of left atrial hypertension to be determined at the discretion of the attending physician, "with the modality of their choice," when a pulmonary artery catheter was not in use, yet provided no further information on the diagnostic modalities used [49]. Therefore, because of the very likely differences in baseline ARDS risk in the various studies' populations, as well as the uncertainty of diagnostic accuracy for ARDS, given the variable criteria used to define it, a meta-analysis was not performed as had been planned *a priori*.

### Clinical outcomes

The results of the studies are shown in Table 2. In the only RCT, a tidal volume of 6 ml/kg predicted body weight (PBW) was associated with an absolute risk reduction of 10.9% for the development of ARDS (number needed to treat of 9.2) when compared with 10 ml/kg PBW. The observational studies included three operating room studies (*n *= 58,419), one of which demonstrated an association between tidal volume and the development of ARDS. However, in this largest trial included (*n *= 53,910) in the review, the tidal volume difference between the two groups was not clinically significant (0.1 ml/kg PBW). Nine of the observational studies were from the ICU (*n *= 8,165), six of which showed tidal volume (mean tidal volume or indexed to PBW) to be an independent predictor of the development of ARDS. Five studies showed a dose-response relation to tidal volume, with the proportion of ARDS increasing with higher tidal volumes. No study reported adverse clinical effects in association with a lower tidal volume.

**Table 2:** Study results

Randomized controlled trial							
**Author, year**	**Time of ARDS onset**	**Incidence of ARDS **	**Experimental ARDS rate**	**Control ARDS rate**	**OR**	***p***	**Comments^a^**

Determann, 2010	1.9 days	8% (*n *=12)	2.6% (*n *= 2)	13.5% (*n *= 10)	5.1 (1.2-22.6)	0.01	Trial stopped early for safety, due to ARDS in control.

**Observational studies**							

**Cohort studies**							

**Author, year**	**Time of ARDS onset**	**Incidence of ARDS **	**VT ARDS Group**	**VT No-ARDS Group**	**OR^b^**	***p***	**Comments**

Gajic, 2004	2.5 days	24% (*n *= 80)	Unknown	Unknown	1.29 (1.12-1.51)	<0.001	Proportion of ARDS increased as VT increased.

Gajic, 2005	Unknown	6.2% (*n *= 205)	670 ml ± 220^c^	620 ml ±110 ^c^	1.26 (1.12-1.40)	<0.001	Proportion of ARDS increased as VT increased.

Kahn, 2006	3 days	27% (*n *= 170)	9.1 ml/kg PBW ±1.8	9.4 ml/kg PBW ±1.5	NS	0.06	ARDS development associated with transfusion, SAH severity, and PRBC transfusion.

Mascia, 2007	2.8 days	22% (*n *=18)	10.4 ml/kg PBW ± 1.1^b^	9.5 ml/kg PBW ±1.0^c^	5.54 (1.54-9.24)	0.008	Proportion of ARDS increased as VT increased.

Yilmaz, 2007║	Unknown	28% (*n *= 60) before protocol; 10% (*n *= 17) after protocol	Unknown	Unknown	1.31 (1.16-1.50)	<0.001	Proportion of ARDS increased as VT increased.

Plurad, 2007	Unknown	8.2% (*n *= 192)	8.6 ml/kg PBW ± 1.7	8.8 ml/kg PBW ± 2.3	NS	0.383	ARDS associated with fluid balance and PRBC transfusion

Iscimen, 2008	5 hours	44% (*n *= 71)	6.9 ml/kg PBW (6.1-7.8)	7.0 ml/kg PBW (6.5-7.9)	NS	0.362	Delayed antibiotics and resuscitation associated with ARDS

Jia, 2008	3.3 days	19% (*n *= 152)	650.5 ml ± 119.7^c^	616.9 ml ± 112.8^c^	1.33 (1.09-1.62)	0.006	Proportion of ARDS increased as VT increased. Transfusion, fluid balance, airway pressures also associated with ARDS. Mean VT in patients that developed ARDS, 623.5 ml

Pasero, 2008	3.7 days	7% (*n *= 14)	Unknown	Unknown	2.04 (1.03-4.05)	0.042	Time of surgery and PEEP also associated with ARDS

Hughes, 2010	Unknown	28% (*n *= 25)	9.0 ml/kg PBW (8.3-10.0)	9.3 ml/kg PBW (8.3-10.1)	1.0 (0.3-3.2)	0.97	Intraoperative fluid >20 ml/kg/h associated with ARDS

Blum, 2011	Unknown	0.2% (*n *= 102)	9.2 ml/kg PBW^c^	9.1 ml/kg PBW^c^	0.77 (0.62-0.95)	0.02	PRBC transfusion strongest predictor of ARDS

**Case-control study**							

**Author, year**	**Time of ARDS onset**	**Incidence of ARDS**	**VT ARDS Group**	**VT No-ARDS Group**	**OR^d^**	***p***	**Comments**

Fernandez-Perez, 2009	Unknown	1.9% (*n *= 83)	8.9 ml/kg PBW ±1.6	8.7 ml/kg PBW ±1.7	1.03 (0.84-1.26)	0.801	Time of surgery, transfusion, and fluid balance associated with ARDS

ARDS, acute respiratory distress syndrome; NS, nonsignificant odds ratio (exact value and confidence interval not reported in original manuscript); OR, odds ratio; PEEP, positive end-expiratory pressure; PRBC, packed red blood cell; SAH, subarachnoid hemorrhage; VT, tidal volume. ^a^Each trial attempted to control for various factors associated with development with ARDS, either with statistical analysis or trial design (for the RCT). ^b^OR for risk of ARDS as a function of tidal volume. ^c^Statistically significant differences existed in VT between groups. ^d^Adjusted OR for VT between ARDS cases and matched controls. ^e^Protocol aimed at VT and transfusion reduction. VT 10.6 ml/kg PBW (9.0-12.1) before protocol versus 7.7 ml/kg PBW (6.7-9.0).

The time of ARDS onset was reported in seven studies, and occurred between days 2 and 4 (5 hours to 3.7 days). The incidence of ARDS was very low in the two operating room studies involving primarily elective procedures: 0.2% and 1.9%. This is in contrast to a much higher incidence in ICU patients, ranging from 6.2% to 44%.

In the studies comparing secondary outcomes between ARDS and non-ARDS groups, the development of ARDS increased mortality and morbidity. ARDS was associated with an absolute increase in mortality of up to 36%, increased ICU and hospital lengths of stay, fewer ventilator-free days, and increased organ failure (Table 3).

**Table 3:** Clinical outcomes comparing ARDS and non-ARDS groups

Outcome	Author, year	ARDS	No ARDS	*p*
Mortality	Kahn, 2006	42%	19%	<0.05

	Fernandez-Perez, 2009	44%	8%	<0.001

	Mascia, 2007	28%	22%	NS

Hospital mortality	Gajic, 2004	34%	24%	0.116

	Gajic, 2005	62%	32%	

	Iscimen, 2008	51%	18%	<0.001

	Fernandez-Perez, 2009	17%	5%	0.004

ICU mortality	Iscimen, 2008	38%	11%	

ICU LOS (days)	Mascia, 2007	25	20	<0.05

	Kahn, 2006	15% increase		<0.05

Hospital LOS (days)	Kahn, 2006	7% increase		NS

	Fernandez-Perez, 2009	17	5	<0.001

VF days	Gajic, 2004	17	22	0.007

	Mascia, 2007	11	16	<0.05

Duration of MV (days)	Kahn, 2006	14	8	<0.001

Renal failure	Kahn, 2006	8%	2%	<0.001

ARDS, acute respiratory distress syndrome; LOS, length of stay; MV, mechanical ventilation; NS, nonsignificant; VF, ventilator free.

## Discussion

In patients who do not have ARDS at the time of endotracheal intubation, the preponderance of evidence implies an association between lower tidal volume and subsequent progression to ARDS. However, this systematic review could yield no definitive recommendations regarding the most appropriate tidal volume strategy in patients without ARDS. Several important caveats exist. An overall lack of data appears in the existing body of literature, as well as a lack of methodologic quality in the data that do exist. Of the 13 trials included in this review, only one was a high quality RCT, whereas the remainder were of observational trial design. None of the observational trials referenced the STROBE guidelines, which are endorsed by 110 journals worldwide [50]. Publication guidelines like STROBE were designed to standardize the reporting of medical research to enhance transparency and minimize variability [51]. An increasing body of evidence indicates that the guidelines do improve the overall quality of research reporting [52].

There was also substantial heterogeneity appeared in the patient populations examined, each carrying perhaps vastly different risk for ARDS progression. For example, relatively healthy patients with a finite exposure to tidal volume risk (operating room cases) are inherently different in risk for progression compared with critically ill ICU patients [53]. Furthermore, even among the studies restricted to patients with longer exposure to tidal volume (ICU patients), the diagnoses leading to mechanical ventilation (Table 1) also carry very different risk for ARDS (e.g. septic shock versus postoperative). Funnel-plot inspection for the assessment of publication bias revealed asymmetry suggestive of publication bias. However, asymmetric funnel plots are not sufficient proof of publication bias. Other potential explanations for asymmetry include study-to-study heterogeneity with the intervention fidelity or outcome assessment. Also, improvement in the usual care in the "control" treatment groups as routine management evolves over time may reduce the observed effect size. It is also possible that an asymmetric funnel plot is the result of chance alone [41]. Finally, differences were noted between studies in how a patient was classified as having developed ARDS, even though each study required fulfillment of accepted consensus criteria [2].

The results of this systematic review do reveal some important trends in the data. First, each study that reported ARDS onset showed that it occurs early in mechanically ventilated at-risk patients. This may reflect the difficulty in defining the precise onset of ARDS retrospectively, but also that many mechanically ventilated patients may have early ARDS that has yet to manifest clinically. This suggests that prevention trials should target the most immediate time after initiation of mechanical ventilation (e.g. in the ED, interhospital transport, and/or the first 24 hours of ICU admission). Endotracheal intubation and initiation of mechanical ventilation is a common occurrence in the ED, yet the effect that ED-based mechanical ventilation may have on outcome has not been studied [54]. However, preenrollment data from ARDS clinical trials showed that early tidal volume did not influence mortality, further highlighting the equipoise on this topic [55].

Second, the incidence of ARDS is very low when tidal volume is studied in low risk patients in the operating room (<2%). This is in contrast to the ICU, where the progression to ARDS appears to be a significant problem (as high as 44%). However, in a cohort of higher-risk surgical patients, higher tidal volumes have been associated with other clinical outcomes, including greater nonpulmonary organ failure and an increase in duration of mechanical ventilation and ICU length of stay [56].

Third, although no definitive conclusions between tidal volume and progression to ARDS can be made, based on this systematic review, several findings in this body of evidence suggest a link between higher tidal volumes and progression to ARDS. Although almost all of the data are observational trials, consistent findings across trial design suggest a cause-effect relation [57]. In the only RCT examining this topic to date, tidal volume was predictive of progression to ARDS. Additionally, the majority of the observational data demonstrate an increased ARDS incidence with larger tidal volumes, which also suggests a causal link [57]. A dose-response relation (seen in five studies), with the proportion of ARDS patients increasing with higher tidal volumes, suggests cause-effect, as does the fact that no findings appeared in the opposite direction (no study showed lower tidal volumes to be harmful).

Fourth, the development of ARDS was associated with many other factors across these trials. These include patient-related (e.g. restrictive lung disease [29]), ventilator-related (e.g. airway pressure [47]), and non ventilator-related (e.g. fluid balance and transfusion [35,45,47,49]) factors. This further highlights that the development of ARDS reflects an interaction between patient risk and treatment variables, which was demonstrated before, and suggests that the most effective ARDS-prevention strategy may involve a multimodal approach (e.g. early resuscitation, fluid management and type, lower tidal volume, high transfusion threshold, and so on) [48,53]. In addition, future ARDS-prevention trials will need to control for each of these confounding variables while assessing patient populations with similar baseline risk of progressing to ARDS.

Finally, the progression to ARDS is associated with significant increase in morbidity and mortality. In all trials directly comparing ARDS versus no-ARDS groups, mortality (up to fivefold increase), length of stay, and organ failure were all increased with the development of the syndrome. Although the best strategy to prevent the progression to ARDS has not been fully elucidated, it is clear that its prevention should be a high priority in clinical care and further research [58].

Important limitations exist in this systematic review. The only RCT was stopped early, after an unplanned interim analysis, because of safety concerns of ARDS development in the 10-ml/kg PBW group [44]. Clinical trials stopped early for benefit are increasing in prevalence, often lack information on why the trial was stopped, and routinely show larger than expected treatment effects [59]. The benefit of clinical trials stopped early, including the trial in this review, should therefore be viewed with caution. However, with 75% of the planned enrollment achieved, and a very significant between-group difference for the primary outcome (*p *= 0.01), confidence is increased that these results are indeed valid.

Second, relatively few studies have examined ARDS prevention and the role of tidal volume in the development of ARDS. Existing studies are limited by their observational trial design, and therefore any attempt to analyze these studies as a group is limited by their individual weaknesses.

Third, a meta-analysis of these data could not be conducted as planned *a priori*, which differs from another systematic review on this topic [60]. The patients were not qualitatively similar and represent a spectrum of patients from those with very little risk for ARDS (e.g. elective surgery patients) to those with very high risk (e.g. septic shock). When contemplating whether a systematic review should become a meta-analysis, heterogeneity is typically first addressed on a macroscopic level of the individual study's design, population, intervention(s), and outcome measures. If sufficient differences exist between individual studies such that lumping them together is comparing "apples and oranges," then a meta-analysis is not appropriate. Systematic review authors should proceed to meta-analysis only if this first level of heterogeneity is assessed and deemed unlikely, an approach that has been previously advocated [61]. We were therefore able to conduct only a qualitative analysis of the data, as conducting a meta-analysis would have served only to homogenize patients artificially. We recognize that these limitations make drawing recommendations difficult based on this systematic review, but with our inclusion of an additional 10 studies (and >7,000 ICU patients) and strict adherence to reporting guidelines, we believe this current review to be the most complete and methodologically sound assessment of the literature to date.

The focus on early mechanical ventilation and ARDS prevention is likely to increase. Recently the National Heart, Lung and Blood Institute (NHLBI) recommended the development of ARDS prevention trials, as well as observational trials of patients without ARDS undergoing prolonged mechanical ventilation [58]. Given the limited treatment options for ARDS, the period of mechanical ventilation immediately after endotracheal intubation is emerging as a critical time window of clinical care and an opportunity for clinical trials to improve outcome [58]. Our results are important in further highlighting knowledge gaps, which may serve in the future design of ARDS prevention trials, and not only call attention to the limitation of the literature, but also highlight potential solutions for clinical trials going forward. This includes RCTs targeting homogeneous patient groups with higher event rates for ARDS progression and early timing of trial enrollment (e.g. immediately after endotracheal intubation in the ED). Factorial trial design aimed at limiting more than one risk modifier for ARDS progression, such as tidal volume and fluid balance, should be considered to maximize efficiency in this research arena [62]. Future research should aim to minimize bias by incorporating publishing guidelines such as the Consolidated Standards of Reporting Trials (CONSORT) for RCTs and the STROBE for observational trials and should have clinically relevant end points, such as progression to ARDS or development of nonpulmonary organ failure as a surrogate for ventilator-associated lung injury [40,63,64]. Finally, to reduce efficiently the preventable heterogeneity and variability in research and clinical care in a sustainable fashion, interventions should be simple, and reproducibility should be enhanced with the aid of implementation science and electronic aids [65-69]. Knowledge of factors influencing a lack of adherence to beneficial therapy in ARDS may aid clinicians in translating research to routine care for prevention of the syndrome as well [65,70]. These trials will ultimately influence clinicians and change practice. Therefore, analyzing critically what has and has not been done is of vital importance.

## Conclusions

Currently, no definitive recommendations can be made on the most appropriate tidal volume strategy in mechanically ventilated patients without ARDS. Data suggest an increased incidence of ARDS with higher tidal volumes, but previous studies are limited by their heterogeneity and high variability in baseline ARDS risk among the patients included. Given the increased mortality and morbidity associated with progression to ARDS, a focus on prevention should remain a high priority for future research and clinical practice.

## Key messages

• The majority of data suggest that higher tidal volumes are causal in the development of ARDS.

• An overall lack of quality data prevents a definitive recommendation for the most appropriate tidal volume in patients with ARDS.

• ARDS occurs early in the course of mechanical ventilation, suggesting that ARDS-prevention trials should occur early in the course of mechanical ventilation, such as in the emergency department.

• The development of ARDS is associated with significant increases in mortality and morbidity, suggesting that ARDS-prevention trials are needed.

## Abbreviations

AECC: American European Consensus Conference; ALI: acute lung injury; ARDS: acute respiratory distress syndrome; CONSORT: Consolidated Standards of Reporting Trials; ED: emergency department; ICU: intensive care unit; MOOSE: Meta-Analysis of Observational Studies in Epidemiology; NCATS: National Center for Advancing Translational Sciences; NCRR: National Center for Research Resources; NHLBI: National Heart: Lung and Blood Institute; NIH: National Institutes of Health; PBW: predicted body weight; PRISMA: Preferred Reporting Items for Systematic Reviews and Meta-Analysis; RCT: randomized controlled trial; STROBE: Strengthening the Reporting of Observational Studies in Epidemiology.

## Competing interests

The authors declare that they have no competing interests.

## Authors' contributions

BF was responsible for conception and design, acquisition of data, analysis, and interpretation of data. He was also responsible for drafting the article, revising it critically for important intellectual content, as well as final approval of the version to be published. NM contributed to the design, acquisition of data, analysis, and interpretation of data. He was also responsible for drafting the article, revising it critically for important intellectual content, as well as final approval of the version to be published. AD contributed to the design, acquisition of data, analysis, and interpretation of data. She was also responsible for drafting the article, revising it critically for important intellectual content, as well as final approval of the version to be published. CC contributed to the design, acquisition of data, analysis, and interpretation of data. He was also responsible for drafting the article, revising it critically for important intellectual content, as well as final approval of the version to be published.

## Supplementary Material

Additional file 1 **PROTOCOL: Search and identification of studies**. This is the search protocol and protocolized strategy, including the inclusion and exclusion criteria that were used capture the studies for this systematic review. Click here for file
