# Supplementary material for: Lower tidal volume at initiation of mechanical ventilation may reduce progression to acute respiratory distress syndrome: a systematic review
Source: Crit Care. 2013 Jan 18;17(1):R11. doi: 10.1186/cc11936 (PMC3983656; doi:10.1186/cc11936)
Supplement: Additional file 1 — PROTOCOL: Search and identification of studies. This is the search protocol and protocolized strategy, including the inclusion and exclusion criteria that were used capture the studies for this systematic review. [file cc11936-S1.DOC]

**PROTOCOL: Search and identification of studies**

*Lower tidal volume at initiation of mechanical ventilation may reduce progression to acute respiratory distress syndrome – a systematic review*

**P**atient/Problem: Mechanically ventilated adult patients without acute respiratory distress syndrome

**I**ntervention: Lower tidal volumes

**C**omparison: Higher tidal volumes

**O**utcome: The development of ARDS

**Clinical question**: In mechanically ventilated patients without acute respiratory distress syndrome, are lower tidal volumes, when compared to higher tidal volumes, associated with a decreased incidence of ARDS?

Search Strategy:

**Acute Lung Injury/Acute Respiratory Distress Syndrome**

"Acute Lung Injury"[Mesh] OR “Acute Respiratory Distress Syndrome” OR "Acute Lung Injury" OR “Acute Lung Injuries” OR "Ventilator-Induced Lung Injury"[Mesh] OR "Ventilator-Induced Lung Injury” OR “Ventilator-Induced Lung Injuries” OR “Ventilator Associated Pneumonia” OR “ventilation induced lung injury” OR “VILI”

**AND**

**Prevention**

Prevent* OR prevention OR prophylax* OR prophylac* OR chemoprevent* OR thwart* OR "ward off" OR "ward-off" OR pre-emptive* OR preemptive* OR chemoprophyla*

**AND**

**Outcome**

(outcome* OR ((treatment* OR protocol*) AND (respond* OR response*)) OR failure* OR mortality OR fatal* OR death OR dead OR deaths OR "passed away" OR demise* OR Recurren* OR progression OR progressed OR relaps* OR growth OR grew OR growing OR regression OR survival OR cure OR cures OR "quality of life" OR qol OR morbidit* OR adverse OR "side effect" OR "side effects" OR event OR events OR nausea OR nauseous OR vomit* OR emesis OR comfort* OR pain OR painful OR painfree OR pain-free OR stress OR analges* OR "Outcome Assessment Health Care "[Mesh] OR "Mortality"[Mesh] OR "mortality "[Subheading] OR "Survival"[Mesh] OR "Survival Analysis"[Mesh] OR "Quality of Life"[Mesh] OR "Pain Measurement"[Mesh] OR "Health"[Mesh] OR "Health Status Indicators"[Mesh] OR "Health Status"[Mesh] )

**AND**

**Adults**

"Young Adult"[Mesh] OR “Young adults” OR “Young Adult” OR "Adult"[Mesh] OR “adults” OR “adult” OR "Middle Aged"[Mesh] OR “Middle age” OR "Aged"[Mesh] OR “Aged” OR Elder* OR "Aged, 80 and over"[Mesh] OR “Oldest Old” OR Nonagenarian* OR Octogenarian* OR Centenarian* OR "Frail Elderly"[Mesh]  OR “Frail Older Adults” OR “Frail Older Adult”

**AND**

**PubMed NOT Animal Studies**

NOT (("Animals"[Mesh]) NOT ("Animals"[Mesh] AND "Humans"[Mesh]))

**Limits: 1967-2011/07/01**

| **Inclusion Criteria** | **Exclusion Criteria** |
| --- | --- |
| **Any language or publication type**  **Age > 17 years**  **Invasive positive pressure ventilation during study period**  **Tidal volume studied or analyzed independently as a predictor variable for outcome**  **Development of ARDS as either primary or secondary outcome measure** | **Acute respiratory distress syndrome at the time of intubation/initiation of mechanical ventilation**  **Non invasive positive pressure ventilation or non intubated patients**  **Intervention primarily for one-lung ventilation**  **Tidal volume studied with another co-intervention (e.g. PEEP change)**  **Non human studies**  **Paper = review, correspondence, or editorial** |

Step 1 (Relevance Screen):

Search MEDLINE, EMBASE, CINHAL, and the Cochrane Library using the above search terms.

BMF and NMM: Screen title and abstract and document the reason for exclusion in electronic database

Step 2:

Identify unpublished data

BMF and NMM: Manually screen reference lists of all review articles from relevance screen

BMF: Search online for details of clinical trials registration (ClinicalTrials.gov)

NMM and BMF: Hand search abstracts from: SCCM, ESICM, ASA, ATS, CHEST, SAEM from 2008 to 2011

BMF and NMM: Manually screen reference lists of all articles to be potentially included from electronic and manual review of review articles

BMF: If unpublished data is found and clarification is needed, contact PI of that study

Step 3:

BMF and NMM: Full review of the remaining manuscripts for agreement. Document in exclusion log why manuscript excluded after full text review

AD: Assess any studies where there is disagreement for inclusion

Step 4:

BMF, NMM, AD: Fill out data abstraction form for final studies included

Step 5: Transfer data from Data Abstraction Form to Table

Step 6: Assess Table for potential for meta-analysis of the data
